# A re-formulation of generalized linear mixed models to fit family data in genetic association studies

**DOI:** 10.3389/fgene.2015.00120

**Published:** 2015-03-31

**Authors:** Tao Wang, Peng He, Kwang Woo Ahn, Xujing Wang, Soumitra Ghosh, Purushottam Laud

**Affiliations:** ^1^Division of Biostatistics, Medical College of WisconsinMilwaukee, WI, USA; ^2^Global Biostatistical Science, Amgen Inc.Thousand Oaks, CA, USA; ^3^Bioinformatics and Systems Biology Core, National Heart, Lung, and Blood InstituteBethesda, MD, USA; ^4^Department of Genetics, Quantitative Sciences, GlaxoSmithKlineKing of Prussia, PA, USA

**Keywords:** family data, generalized linear mixed models (GLMM), genetic correlation, genetic variance components, random genetic effects, re-parameterization, Cholesky decomposition, Bayesian methods

## Abstract

The generalized linear mixed model (GLMM) is a useful tool for modeling genetic correlation among family data in genetic association studies. However, when dealing with families of varied sizes and diverse genetic relatedness, the GLMM has a special correlation structure which often makes it difficult to be specified using standard statistical software. In this study, we propose a Cholesky decomposition based re-formulation of the GLMM so that the re-formulated GLMM can be specified conveniently via “proc nlmixed” and “proc glimmix” in SAS, or OpenBUGS via R package BRugs. Performances of these procedures in fitting the re-formulated GLMM are examined through simulation studies. We also apply this re-formulated GLMM to analyze a real data set from Type 1 Diabetes Genetics Consortium (T1DGC).

## 1. Introduction

Generalized linear mixed model (GLMM) provides a rich class of statistical models to model correlated data with responses from the exponential family of distributions including Gaussian, Binomial, Poisson, etc. (see McCulloch and Searle, 2001). The mixed model approach, which is also called the variance component approach, has long been used in genetic studies to estimate genetic parameters, predict breeding values and model correlated family or pedigree data (Henderson, 1963, 1975; Amos, 1994; Falconer and Mackay, 1996; Almasy and Blangero, 1998; Abecasis et al., 2000; Sham and Purcell, 2001). Due to the diverse genetic correlation structures among families or pedigrees, currently fitting this type of GLMM often relies on special genetic software packages, such as SOLAR (Almasy and Blangero, 1998), Multic (de Andrade et al., 1998, 2006). More recently, as an extension of the R package *lme4*, Vazquez et al. (2010) also developed an R package *pedigreemm* following the method of Harville and Callanan (1989). *Pedigreemm* can handle the additive genetic correlation among all sampled individuals via a Cholesky decomposition of the coancestry (or so-called numerator relationship) matrix. However, despite the popularity of these software packages, they often lack many options such as choosing different algorithms for maximizing the likelihood or specifying various particular type of the covariance structures that are available in standard statistical software packages such as procedures “proc mixed,” “proc glimmix,” and “proc nlmixed” in SAS (SAS Institute, Inc.) or OpenBUGS in R (e.g., via R package BRugs). One major obstacle in using these standard software packages to fit the GLMM is the requirement of the same correlation structures across all the families (or clusters). A few recent studies also suggested fitting the GLMM by using SAS (Feng et al., 2009; Wang et al., 2011). But it appears that these studies have only considered the cases where all the families had the same genetic correlation structure.

The genetic correlation structure among family members for additive effects, dominance and epistasis has been well described (see Lynch and Walsh, 1998). In this study, for the special type of GLMM that models family data with varied family sizes or diverse genetic relatedness, we propose a Cholesky decomposition based re-formulation of the GLMM so that the re-formulated GLMM can be specified conveniently when using some standard statistical software packages. First, a standard GLMM is presented, which can account for the genetic correlation among family members. We briefly discuss the identifiability issue of the variance components from their possible confounding perspective in the GLMM. Next, we explain how the GLMM can be re-formulated into a GLMM with random regression coefficients of equal variances. Assuming that there is no genetic correlation between different families, we start by applying separate Cholesky decompositions on the genetic kinship matrices within each family. Then we stack these Chelosky decomposition matrices from different families into one column and treat the columns of the stacked matrix as fixed covariates. Unlike the regular Cholesky decomposition performed on the whole covariance matrix of the random genetic effects (e.g., Vazquez et al., 2010), here the stacked matrix has its column size being the maximum family size instead of the summation of all family sizes. With the reduced number of columns, this re-formulated GLMM can then be specified conveniently in “proc nlmixed” or “proc glimmix” using SAS, and OpenBUGS via R package BRugs. We provide detailed codes on fitting this re-formulated GLMM by using either “proc nlmixed” or “proc glimmix” in SAS, and OpenBUGS with R (via R package BRugs). The performances of these procedures on fitting the re-formulated GLMM are also examined through some simulation studies. Finally, we apply this re-formulated GLMM to a real data set from Type 1 Diabetes Genetics Consortium (T1DGC).

## 2. Methods

### 2.1. A generalized linear mixed model (GLMM)

Suppose that we have a randomly collected sample of *N* families from a study population. In the *i*-th family of size *n_i_*, let *y_ij_* be a (binary or continuous) response variable for a disease phenotype; *z_ij_* be some fixed environmental covariates that need to be adjusted for; *g_ij_* denote the observed genotypes at certain targeted genetic marker loci, for family members *j* = 1, 2, ···, *n_i_* and *i* = 1, 2, ···, *N*. To model the family data and test for the association of *g_ij_* with the phenotypic response *y_ij_*, we need to account for the genetic correlation among family members induced by identity-by-descent (IBD) alleles shared by the family members at some putative disease susceptible loci (DSL). In addition, family members may share certain common environmental factors which could also contribute to the disease phenotypes. Let *v_ij_* denote the random genetic effect from those putative (unobserved) DSL on the phenotypic response *y_ij_*, and *e_i_* be the shared environmental effect such as diets for members in a family *i*. Define μ_*ij*_ = E(*y_ij_*|*v_ij_*, *e_i_*). Then a generalized linear mixed model (GLMM) to model the family data can be written as

(1) $$\{\begin{array}{l} y_{ij}|v_{ij},e_{i}\sim f_{y_{ij}|v_{ij},e_{i}}(y|v_{ij},e_{i}),\;j=1,\cdots ,n_{i}\\ \text{E}(y_{ij}|v_{ij},e_{i})\;=\;\mu_{ij}\\ g(\mu_{ij})\;=m+z_{ij}\alpha +x(g_{ij})\beta +v_{ij}+e_{i},\;\;j=1,\cdots ,n_{i}\\ v_{i}\;=\;{\left(v_{i1},\cdots ,v_{in_{i}}\right)}^{T}\sim N(0,\Sigma_{i})\\ e_{i}\sim N(0,\sigma_{c}^{2}),e_{i}⊥v_{i} \end{array}$$

where *g*(·) is a known link function, *m* is an intercept for the baseline, α is a p-dimensional vector of parameters for the fixed effects of environmental covariates *z_ij_*, *x*(*g_ij_*) is a q-dimensional vector with its components being defined by certain coding functions for marker genotypes (see Wang, 2011), and β is a q-dimensional vector of parameters for the fixed genetic effects contributed by the observed genotypes *g_ij_*. Typically, the random effects {**v_i_**, *i* = 1, ···, *n*} from different families are assumed to be independent. Given the random effects **v_i_** and *e_i_*, *y_ij_*, *j* = 1, ···, *n_i_*, are also assumed to be conditionally independent. As each *v_ij_* represents an aggregated polygenic effect from multiple putative DSLs, *v_ij_* tends to be normally distributed based on the Central Limit Theorem. Therefore, **Σ_i_** denotes the genetic covariance matrix among the *g*-transformed conditional means *g*(μ_*i*1_), ···, *g*(μ*_in_i__*), which is often induced by identity-by-descent (IBD) alleles shared by the *i*-th family members at the putative DSL.

For a quantitative phenotypic trait, the link function *g* is often chosen as an identity function (i.e., *g*(*x*) = *x*). Assuming that there is no inbreeding between parents, it has been known that the genetic covariance between a pair of relatives *j*, *k* within a family *i* can be expressed as Kempthorne (1955), Amos (1994), Lynch and Walsh (1998), and Yu et al. (2006)

(2) $$\text{Cov}(v_{ij},v_{ik})=\;2\phi_{jk}\sigma_{A}^{2}+\delta_{jk}\sigma_{D}^{2}$$

where σ^2^_*A*_ and σ^2^_*D*_ are the so-called additive and dominant genetic variance components, which are contributed by the additive allelic effects and allelic interactions from those unknown DSL, respectively; ϕ_*jk*_ and δ_*jk*_ are the so-called kinship and double coancestry coefficients between the two relatives. Similarly, for a general link function *g*, we can also define σ^2^_*A*_ and σ^2^_*D*_ as the additive and dominant variance components contributed by the allelic effects and allelic interactions from those unknown DSL to the variation of the *g*-transformed conditional means *g*(μ_*ij*_). Let μ**_i_** = (μ_*i*1_, ···, μ*_in_i__*). From model Equations (1), (2), we have

$$\text{Cov}(g(\mu_{i}))=\;2\Phi_{i}\sigma_{A}^{2}+\Delta_{i}\sigma_{D}^{2}+\sigma_{c}^{2}{\text{J}}_{n_{i}}$$

where J*_n_i__* is a *n_i_* × *n_i_* matrix of 1's, Φ_*i*_ = (ϕ_*jk*_) and Δ_*i*_ = (δ_*jk*_) are *n_i_* × *n_i_* kinship and double coancestry matrices, respectively, for the *i*-th family members. In the absence of inbreeding, the expected kinship and double coancestry coefficients for various common relatedness have been well established (e.g., see Table 7.1 in Lynch and Walsh, 1998). In practice, the actual kinship and double coancestry coefficients could deviate from their expected values because the realized IBD status could vary for a particular pair of family members due to the randomness in their parents' inheritance segregation. Two parents could also be related to each other due to possible inbreeding from their common ancestry. Therefore, the genetic covariance matrix **Σ_i_** = 2Φ_*i*_σ^2^_*A*_ + Δ_*i*_ σ^2^_*D*_ may have varied sizes across different families. For families with the same size and relatedness, their actual kinship and double coancestry matrices could also be different. Nowadays, with the high density of genome-wide genetic markers available, such as single nucleotide polymorphism (SNPs), it is possible to estimate the actual genome-wide kinship and double coancestry coefficients among family members using external programs such as PLINK (Purcell et al., 2007). The genome-wide kinship matrix can also be estimated as half of the genomic relationship matrix (see VanRaden, 2008).

The above GLMM provides a rich class of statistical models, which is applicable to both quantitative and qualitative traits. Depending on the family structures, however, not all the variance components in a GLMM are always estimable. For example, for a quantitative trait with *g*(·) being the identity link function, the GLMM becomes a linear mixed model (LMM).

$$y_{ij}\;=m+z_{ij}\alpha +x(g_{ij})\beta +v_{ij}+e_{i}+\epsilon_{ij},$$

where *i* = 1, …, *N*, *j* = 1, … *n_i_*, ϵ_*ij*_ ~ *N*(0, σ^2^) are the model residuals, with σ^2^ being the residual variance of random effects from other risk factors not captured by *z_ij_*, *g_ij_*, *v_ij_*, and *e_i_*. When each family comprises two parents and one offspring, the expected Δ_*i*_ = *I*_3_, which is a 3 × 3 identity matrix for any family *i*. We have

$$\text{Cov}(y_{i})\;=2\Phi_{i}\sigma_{A}^{2}+(\sigma_{D}^{2}+\sigma^{2})I_{3}+\sigma_{c}^{2}J_{3},\;i=1,\cdots ,N$$

Thus, the dominant variance component σ^2^_*D*_ and the residual variance σ^2^ are completely confounded. Similarly, if each family consists of only siblings (e.g., in a sib-pair design), then

$$\begin{array}{l} \text{V}(y_{ij})=\;\sigma_{A}^{2}+\sigma_{D}^{2}+\sigma_{c}^{2}+\sigma^{2},\;i=1,\cdots ,N\\ \text{Cov}(y_{ij},y_{ik})\;=\;\frac{1}{2}\sigma_{A}^{2}+\frac{1}{4}\sigma_{D}^{2}+\sigma_{c}^{2},\;j\neq k \end{array}$$

In this case, we cannot distinguish the four variance components from each other unless some of them are negligible (e.g., assuming σ^2^_*D*_ = σ^2^_*c*_ = 0). For a sample of unrelated individuals, it is also easy to see that all the four variance components σ^2^_*A*_, σ^2^_*D*_, σ^2^_*c*_, and σ^2^ are inseparable.

### 2.2. In fitting the GLMM

The main goal in fitting GLMM Equation (1) is to make statistical inference on the fixed effects α, β as well as assessing the variance components σ^2^_*A*_, σ^2^_*D*_, and σ^2^_*c*_. Based on model Equation (1), the full likelihood is

$$\begin{array}{l} L(\alpha ,\beta |\{y_{ij},z_{ij},g_{ij}\})\;=\;\prod_{i=1}^{N}\int_{v_{i}}\int_{e_{i}}f_{y_{i}|v_{i},e_{i}}(y_{i}|v_{i},e_{i})\;f_{v_{i}}(v_{i})\\ \;f_{e_{i}}(e_{i})\;de_{i}dv_{i} \end{array}$$

We need to calculate a multi-dimensional integration for each family, which in most cases cannot be analytically evaluated in closed forms. Various methods in fitting a standard GLMM have been proposed based on either numerical approximations to the integrations or a linearization of the regression model. Traditional methods for numerical integral approximation include Laplace approximation (Wolfinger, 1993), the adaptive Gauss-Hermite quadrature (Pinheiro and Bates, 1995), Monte Carlo integration, and Bayesian method via Markov chain Monte Carlo. The model linearization is often made via Taylor expansion on the inverse of the nonlinear link function *g*(·), based on which the pseudo-likelihood or restricted pseudo-likelihood for optimization can then be derived (Wolfinger and O'Connell, 1993). It has been known that the numerical integral approximations could become computationally intractable when a large number of random effects are involved. On the other hand, the model linearization approach could encounter severe unconvergence problems especially for binary outcomes with small cluster (family) sizes.

In practice, several common statistical software packages are available in fitting a standard GLMM. These include but not limited to “proc nlmixed” and “proc glimmix” procedures in SAS, and OpenBUGS for Bayesian approach. The “proc nlmixed” mainly conducts integral approximations using an adaptive Gauss-Hermite quadrature as default, and then directly maximizes the approximately integrated likelihood. In contrast, the “proc glimmix” primarily performs several model linearization based pseudo-likelihood methods, although it can also fit the GLMM using Laplace or adaptive Gauss-Hermite quadratures for integral approximations (see documentations supported by SAS Institute Inc, Raleigh, NC). In addition, the model fitting algorithm in R package “lme4” consists of an iteration between two sub-optimization procedures. One is to determine the conditional modes of the fixed and random effects, given the current deviance and variance components using penalized iteratively re-weighted least squares (PIRLS). The other is to obtain MLE of the deviance and variance components based on a profile likelihood from Laplace approximation, given the current conditional modes of the fixed and random effects (Bates et al., 2012). Nonetheless, these software packages typically require the random effects to have the same covariance (or correlation) structures across all the clusters. When families have different sizes or varied kinship or double coancestry matrices, it is difficult to directly specify GLMM Equation (1) using these software packages. It should be pointed out that the R package *pedigreemm* can fit a GLMM to family data with complex family structures without separating families into clusters. It also allows individuals from different families to be correlated. Here, we focus on fitting GLMM (1) using “proc nlmixed” and “proc glimmix” procedures in SAS, or OpenBUGS as an alternative choice.

To deal with different families sizes or varied kinship or double coancestry matrices, we apply a Cholesky decomposition based re-parameterization of the random genetic effects **v_i_** to re-formulate GLMM Equation (1) into a GLMM with random regression coefficients. First, we apply separate Cholesky decompositions on the kinship and double coancestry matrices: 2Φ_*i*_ = *L*_Φ*i*_*L*^*T*^_Φ*i*_, Δ_*i*_ = *L*_Δ*i*_*L*^*T*^_Δ*i*_, for *i* = 1, ···, *N*. We then re-parameterize the random genetic effects within family *i* as **v_i_** = *L*_Φ*i*_**a_i_** + *L*_Δ*i*_**d_i_**, where **a_i_** = (*a*_*i*1_, ···, *a_ir_i__*)^*T*^ ~ *N*(0, σ^2^_*A*_*I_r_i__*) with *r_i_* = *rank*(Φ_*i*_), and **d_i_** = (*d*_*i*1_, ···, *d_is_i__*)_*T*_ ~ *N*(0, σ^2^_*D*_*I_s_i__*) with *s_i_* = *rank*(Δ_*i*_). Besides, we assume that **a_i_**'s are independent of **d_i_**'s. By replacing **v_i_** by *L*_Φ*i*_**a_i_** + *L*_Δ*i*_**d_i_** in model Equation (1), we have

$$\text{Cov}(v_{i})\;=\text{ Cov}(L_{\Phi i}a_{i}+L_{\Delta i}d_{i})\;=2\Phi_{i}\sigma_{A}^{2}+\Delta_{i}\sigma_{D}^{2}=\Sigma_{i}$$

Note that the random effects **a_i_** and **d_i_** are orthogonal within families as well as across families, even though the dimension of these random effects may still vary from family to family because the families may have different sizes.

Next, to deal with possible different family sizes, let *r* = *max_i_*{*r_i_*} and *s* = *max_i_*{*s_i_*} be the maximum number of columns in {*L*_Φ*i*_, *i* = 1, ···, *N*} and {*L*_Δ*i*_, *i* = 1, ···, *N*}, respectively. For those families with *r_i_* < *r* (or *s_i_* < *s*), we further expand their design matrices *L*_Φ*i*_ (or *L*_Δ*i*_) to r (or s) columns by adding *r* − *r_i_* (or *s* − *s_i_*) columns of 0's at the right end (or any places). Then, we obtain a re-formulated GLMM with all the families having the same dimension *r* (or *s*) for their random effects **a_i_** (or **d_i_**) as the following.

$$g(\mu_{i})\;=1_{n_{i}}m+Z_{i}\alpha +X(g_{i})\beta +L_{\Phi i}a_{i}+L_{\Delta i}d_{i}+1_{n_{i}}e_{i}$$

where *Z_i_* = (*z*_*i*1_, ···, *z_in_i__*)^*T*^, *X*(**g_i_**) = (*x*(*g*_*i*1_), ···, *x*(*g_in_i__*))^*T*^, **a_i_** ~ *N*(0, σ^2^_*A*_*I_r_*), **d_i_** ~ *N*(0, σ^2^_*D*_*I_s_*) and *e_i_* ~ *N*(0, σ^2^_*c*_). Finally, we construct the design matrix *L*_Φ_ of **a_i_** from *L*_Φ*i*_, *i* = 1, ···, *N*, by stacking them one above the other; and similarly build the design matrix *L*_Δ_ of **d_i_** by stacking *L*_Δ*i*_, *i* = 1, ···, *N*, one above the other. Now, the columns of the two matrices *L*_Φ_ and *L*_Δ_ can be treated as (*r* + *s*) ordinary fixed continuous covariates with **a_i_**'s and **d_i_**'s being their random regression coefficients. Within each family, all the family members share the same set of slopes. Across different families, the **a_i_**, *i* = 1, ···, *N* (or **d_i_**, *i* = 1, ···, *N*), are independent but share the same variance component σ^2^_*A*_ (or σ^2^_*D*_).

The re-formulated GLMM above can be easily specified by “proc nlmixed” or “proc glimmix” procedures in SAS. With “proc glimmix,” we can use three separate “random” commands “random *e*/subject = famid,” “random *La*_1_ … *La*_*r*_/subject = famid type = TOEP(1);” and “random *Ld*_1_ … *Ld_s_*/subject = famid type = TOEP(1);” to specify the correlation structures for the random effects *e_i_*, **a_i_**, and **d_i_**, respectively. Here, *La*_1_,…, *La_r_* represent the columns of the stacked matrix *L*_Φ_; *Ld*_1_ … *Ld_s_* denote the columns of the stacked matrix *L*_Δ_. The option “TOEP(1)” can force all the elements in **a_i_** (or **d_i_**) to share the same variance component σ^2^_*A*_ (or σ^2^_*D*_). For “proc nlmixed,” currently it only allows to have one “random” command. But we can specify a joint multivariate normal distribution for *e_i_*, **a_i_**, and **d_i_** via “random *e a*_1_ … *a_r_ d*_1_ … *d_s_* ~ normal(mu, v) subject = famid,” where v is a diagonal matrix with one σ^2^_*c*_, *r* elements of σ^2^_*A*_'s and *s* elements of σ^2^_*D*_'s on its diagonal. As an example, the SAS codes for specification of a GLMM using both “proc glimmix” and “proc nlmixed” for families with two parents and two full sibs (i.e., *r* = *s* = 4) are provided in Appendices A, B (Supplementary Material), respectively. We also explored using the R package *lme4* to fit the re-formulated GLMM. But it appears that the functions “lmer” and “glmer” provided in *lme4* do not have an option that can force all the elements in **a_i_** (or **d_i_**) to share the same variance component σ^2^_*A*_ (or σ^2^_*D*_).

This re-formulation also makes it more convenient to fit GLMM Equation (1) using the Markov chain Monte Carlo (MCMC) based Bayesian approach. We use R package BRugs to get access to OpenBUGS software (Christensen et al., 2011), which has the Bayesian approach implemented. It is noticed that OpenBUGS has a weak support for matrix operations. In the original GLMM Equation (1), the genetic covariance matrix **Σ_i_** = **2Φ_i_σ^2^_A_** + **Δ_i_ σ^2^_D_** involves two unknown variance components σ^2^_*A*_ and σ^2^_*D*_. As a result, we cannot directly specify the covariance matrix **Σ_i_** in OpenBUGS. With the re-formulated GLMM, we can pass *L*_Φ_ and *L*_Δ_ as fixed covariates to OpenBUGS with **a_i_** and **d_i_** being their random regression coefficients. Since the Bayesian approach often treats all the model parameters as random, it appears especially suitable for fitting the re-formulated GLMM. We can also extract from the MCMC the posterior distributions of random effects for each individual, which allow us to assess the variation contribution from the putative random genetic effects {*v_ij_*} to the total variance of {*g*(μ_*ij*_)}. The R codes for specification of a re-formulated GLMM using BRugs + OpenBUGS are also provided in Appendix C (Supplementary Material).

## 3. Simulation study

In this section, we examine the performances of procedures “proc nlmixed” and “proc glimmix” in SAS (version 9.3) as well as R packages BRugs + OpenBUGS in fitting the re-formulated GLMM through simulation. We consider three biallelic genetic markers with alleles “0” or “1,” and one fixed explanatory covariate *Z* ~ Bernoulli(0.5). The three genetic markers are assumed to be unlinked (i.e., independent) and have allele frequencies *p*_1_ = 0.5, *p*_2_ = 0.2, *p*_3_ = 0.1 for alleles “1” at locus 1, 2, and 3, respectively. We first generate a pairs of haplotypes independently for each parent, where each haplotype is comprised of three alleles randomly generated from *Bernoulli*(*p_j_*) for *j* = 1, 2, and 3, respectively. Each child inherits one haplotype from father, and the other from mother. The haplotype from father (or mother) consists of three alleles with each allele being selected from the two paternal (or maternal) alleles at the same locus with 50% chance. In case where a family has more than one child, the above random process is repeated for each child independently. For simplicity, we use the expected values to construct the kinship and double coancestry matrices for each family.

We first consider simulating quantitative traits from a LMM. Let **g**_*ij*_ = (*g*_*ij*1_, *g*_*ij*2_, *g*_*ij*3_) be the genotypes of the *j*-th subject in family *i*, where *g_ijk_* ϵ {0, 1, 2} counts the number of alleles “1” at locus *k* = 1, 2, 3. The quantitative trait values are simulated from the following LMM.

$$\begin{array}{l} y_{ij}=m+z_{ij}\alpha +x(g_{ij1})\beta_{1}+x(g_{ij2})\beta_{2}+x(g_{ij3})\\ \;\beta_{3}+v_{ij}+e_{i}+\epsilon_{ij},i=1,\ldots ,n,\;j=1,\ldots n_{i} \end{array}$$

where *x*(*g_ijk_*) = (*x_a_*(*g_ijk_*), *x_d_*(*g_ijk_*))β_*k*_ with *x_a_*(*g_ijk_*) = *g_ijk_*, *x_d_*(*g_ijk_*) = 1 (or 0) if *g_ijk_* = 2 (or otherwise) based on the allele-coding (see Wang, 2011), and β_*k*_ = (β_*ka*_, β_*kd*_)^*T*^ being the effects of marker *k* for *k* = 1, 2, 3. We further set σ^2^_*c*_ = σ^2^ = σ^2^_*A*_ = 1 and σ^2^_*D*_ = 0.5. The other true values of parameters are listed in Table 1. Each simulation data set contains *n*_1_ families with two parents and one child and *n*_2_ families with two parents and two children. We consider three cases: (a) *n*_1_ = 500, *n*_2_ = 500; (b) *n*_1_ = 1000, *n*_2_ = 1000; (c) *n*_1_ = 0, *n*_2_ = 2000.

**Table 1:** **The true values of model parameters in simulation**.

**Parameter**	**Definition**	**True value**
*n*_1_	The number of families with one child	0, 500, 1000
*n*_2_	The number of families with two children	500, 1000, 2000
*m*	The intercept	10
α	The fixed effect of Z	2
β_1_	The genetic effects of marker 1	(1, -2)
β_2_	The genetic effects of marker 2	(1, 0)
β_3_	The genetic effects of marker 3	(1, -1)

For each simulation data set, we fit the GLMM by using two methods: (1) adaptive Gaussian quadrature (AGQ) via “proc nlmixed” in SAS; (2) Bayesian approach via BRugs + OpenBUGS. Based on 200 simulation data sets, the parameter estimates are summarized in **Table 3**. We also explored using “proc glimmix” to fit the simulation data but abandoned it due to some severe unconvergence problems. In running BRugs + OpenBUGS, we choose the following priors for initialization of the parameters: *m* ~ *N*(0, 10), α ~ *N*(0, 10), β_*ka*_ ~ *N*(0, 10) and β_*kd*_ ~ *N*(0, 10) for *k* = 1, 2, 3, τ_*a*_ ~ *Gamma*(1, 1), σ_*D*_ ~ *Uniform*(0, 5), τ_*c*_ ~ *Gamma*(1, 1), and τ ~ *Gamma*(1, 1), where τ_*a*_ = 1/σ^2^_*A*_, τ_*d*_ = σ^2^_*D*_, τ_*c*_ = 1/σ^2^_*c*_, and τ = 1/σ^2^. We use the first 10,000 updates as burn-in, and another 10,000 updates to estimate parameters as posterior means. The parameter estimates and their standard deviations (SD) from the 200 simulations are summarized in Table 2. For each simulation data, we also calculate the length of the 95% confidence (or probability) interval for each parameter in running “proc nlmixed” (or BRugs + OpenBUGS). The average length of these intervals and their coverage rate for each true parameter value from the 200 simulations are summarized in Table 3.

**Table 2:** **Mean and standard deviation (SD) of the parameter estimates from 200 simulations for linear mixed models**.

**No. families**	**(a) *n*_1_ = 500, *n*_2_ = 500**		**(b) *n*_1_ = 1000, *n*_2_ = 1000**		**(c) *n*_1_ = 0, *n*_2_ = 2000**	
**Parameters**	**AGQ**	**Bayesian**	**AGQ**	**Bayesian**	**AGQ**	**Bayesian**
*m*	10.01 (0.07)	10.00 (0.07)	10.00 (0.06)	9.99 (0.05)	10.00 (0.05)	10.00 (0.05)
α	2.00 (0.06)	2.01 (0.06)	2.00 (0.04)	2.00 (0.04)	2.00 (0.03)	2.00 (0.03)
β_1*a*_	1.00 (0.07)	1.00 (0.07)	1.00 (0.05)	1.00 (0.05)	1.00 (0.05)	1.00 (0.05)
β_1*d*_	−2.00 (0.11)	−2.00 (0.11)	−2.01 (0.08)	−2.00 (0.07)	−1.99 (0.07)	−2.00 (0.07)
β_2*a*_	0.99 (0.06)	1.01 (0.06)	1.01 (0.05)	1.00 (0.05)	1.00 (0.04)	1.01 (0.05)
β_2*d*_	0.04 (0.17)	−0.01 (0.18)	−0.00 (0.11)	−0.02 (0.11)	−0.00 (0.10)	0.00 (0.11)
β_3*a*_	1.00 (0.08)	1.00 (0.08)	1.00 (0.05)	1.00 (0.05)	1.00 (0.05)	1.00 (0.05)
β_3*d*_	−0.98 (0.30)	−0.97 (0.33)	−1.00 (0.21)	−0.99 (0.21)	−1.00 (0.21)	−0.98 (0.17)
σ^2^_*A*_	1.01 (0.18)	0.98 (0.18)	1.01 (0.12)	1.00 (0.12)	1.00 (0.11)	1.00 (0.11)
σ^2^_*D*_	0.54 (0.36)	0.46 (0.31)	0.53 (0.30)	0.49 (0.27)	0.51 (0.22)	0.49 (0.22)
σ^2^_*c*_	0.99 (0.11)	1.01 (0.12)	0.99 (0.08)	1.00 (0.08)	0.99 (0.08)	1.01 (0.08)
σ^2^	0.95 (0.36)	1.06 (0.32)	0.97 (0.31)	1.02 (0.27)	0.99 (0.23)	1.03 (0.23)

**Table 3:** **The average length of the 95% confidence (or probability) intervals and the coverage rate for true parameters from 200 simulations for linear mixed models**.

**No. families**	**(a) *n*_1_ = 500, *n*_2_ = 500**		**(b) *n*_1_ = 1000, *n*_2_ = 1000**		**(c) *n*_1_ = 0, *n*_2_ = 2000**	
**Parameters**	**AGQ**	**Bayesian**	**AGQ**	**Bayesian**	**AGQ**	**Bayesian**
*m*	0.31, 96.0%	0.31, 96.0%	0.22, 94.5%	0.22, 96.0%	0.21, 96.0%	0.21, 96.5%
α	0.21, 94.5%	0.21, 94.5%	0.15, 95.0%	0.15, 97.0%	0.14, 96.0%	0.14, 95.0%
β_1*a*_	0.28, 96.0%	0.28, 94.0%	0.20, 95.0%	0.20, 96.5%	0.18, 95.0%	0.18, 96.5%
β_1*d*_	0.43, 95.5%	0.43, 95.0%	0.30, 93.5%	0.30, 97.0%	0.28, 93.0%	0.28, 92.0%
β_2*a*_	0.26, 97.5%	0.26, 96.5%	0.18, 95.5%	0.18, 94.5%	0.17, 97.0%	0.17, 90.5%
β_2*d*_	0.67, 93.5%	0.67, 92.0%	0.47, 96.0%	0.47, 97.5%	0.44, 96.0%	0.44, 96.0%
β_3*a*_	0.32, 95.0%	0.32, 96.5%	0.23, 98.5%	0.23, 96.5%	0.21, 95.5%	0.21, 94.5%
β_3*d*_	1.22, 96.5%	1.20, 92.5%	0.85, 94.5%	0.85, 97.0%	0.78, 95.0%	0.78, 98.5%
σ^2^_*A*_	0.68, 94.5%	0.68, 92.0%	0.49, 96.0%	0.48, 95.0%	0.47, 97.0%	0.46, 95.0%
σ^2^_*D*_	1.61, 87.0%	0.77, 74.0%	1.16, 89.5%	0.66, 75.0%	0.84, 93.5%	0.62, 84.0%
σ^2^_*c*_	0.44, 94.5%	0.44, 95.0%	0.32, 94.5%	0.31, 94.0%	0.31, 96.0%	0.31, 94.0%
σ^2^	1.54, 87.0%	0.87, 74.5%	1.12, 88.5%	0.72, 75.5%	0.86, 95.0%	0.67, 85.0%

From Table 2 we can see that both methods can provide reasonable estimates of the fixed effects α, β_*ka*_, and β_*kd*_ (*k* = 1, 2, 3) as well as the variance components with improved accuracy as sample size increases. As expected, the estimate of allelic interaction β_*kd*_ has a larger SD than that of the additive allelic effect β_*ka*_ at each locus *k*, for *k* = 1, 2, 3. The estimates of σ^2^_*D*_ and σ^2^ are slightly biased, which could be caused by the potential confounding between these two variance components. Besides, it appears that “proc nlmixed” tends to over-estimate the dominant variance σ^2^_*D*_ and under-estimate the residual variance σ^2^. Meanwhile, the Bayesian method heads to the opposite way. From Table 3 the coverage probabilities are close to the nominal level of 95% for most parameters except σ^2^_*D*_ and σ^2^. The average lengths of the 95% confidence (or probability) intervals for σ^2^_*D*_ and σ^2^ are also greater than the ones for other two variance components. In addition, the average length of the 95% confidence (or probability) intervals for β_3*d*_ is substantially larger than that for β_1*d*_ and β_2*d*_, which is likely caused by the fact that the allele “1” at marker locus 3 is rare and the homozygous genotypes “11” at marker locus 3 are much less present in a simulation data set than those at the other two loci.

Overall, the results from “proc nlmixed” and BRugs + OpenBUGS are quite comparable in all three cases. As we have mentioned before, the variance components σ^2^ and σ^2^_*D*_ are confounded in single-child families, and only two-child families are informative for distinguishing them. In case (b), where there is an increased number of 2-child families, the estimates of these two variance components are improved with both methods. In case (c), the accuracy in estimates of these two variance components is further improved. In terms of the computational speed, it appears that the “proc nlmixed” runs much faster than BRugs + OpenBUGS. We ran BRugs + OpenBUGS on one laptop installed with Intel(R) Core(TM)i7-3520M CPU @ 2.90GHz. It took about 19 h, 50 h and 38 min, and 59 h and 15 min of CPU time to complete the 200 simulations (including data generation and MCMC iterations) in cases (a), (b), and (c), respectively. The “proc nlmixed” procedure in SAS was performed on a UNIX workstation which has a compatible speed with the laptop, and it took about 2 and a half hours, 6 and a half hours, and 7 h 18 min of CPU time to complete the 200 simulations in cases (a), (b), and (c), respectively. By contrast, the Bayesian approach can provide the posterior distributions for all the parameters rather than just the modes (MLE) and their variance or covariance estimates. When σ^2^ and σ^2^_*D*_ are almost completely confounded, we found that the “proc nlmixed” may give unreliable estimates of the model parameters or encounter unconvergence problem caused by the nearly singular Hessian matrix, while the Bayesian approach can still provide reasonable estimates at least for other model parameters except σ^2^ and σ^2^_*D*_.

We also consider binary traits and simulate phenotypic values from the following mixed logistic regression model.

$$\begin{array}{l} \text{logit}P(y_{ij}\;=1|z_{ij},g_{ij1},g_{ij2},g_{ij3},v_{ij},e_{i})\;=m+z_{ij}\alpha \\ \;+\;x(g_{ij1})\beta_{1}+x(g_{ij2})\beta_{2}+x(g_{ij3})\beta_{3}+v_{ij}+e_{i}, \end{array}$$

for *i* = 1, …, *n* and *j* = 1, …, *n_i_*. In order to have enough number of events, we choose *m* = −3. Meanwhile, we keep the same true values as before for other model parameters. We explored various options on using “proc nlmixed” and “proc glimmix” but failed to fit the above model appropriately due to severe unconvergence problems. By contrast, BRugs + OpenBUGS can still provide reasonable estimates for most of the model parameters. Using the similar priors in the previous setting, 10,000 burn-in and 10,000 updates for parameter estimation, we compute the means and standard deviations (SD) of the parameter estimates and the average lengths (AL) and coverage rates (CR) of the 95% probability intervals from 200 simulations as summarized in Table 4.

**Table 4:** **Means (SD) of the parameter estimates and AL (CR) of the 95% probability intervals from 200 simulations for mixed logistic regression models**.

**No. families**	**(a) *n*_1_ = 500, *n*_2_ = 500**		**(b) *n*_1_ = 1000, *n*_2_ = 1000**		**(c) *n*_1_ = 0, *n*_2_ = 2000**	
**Parameters**	**Mean (SD)**	**AL (CR)**	**Mean (SD)**	**AL (CR)**	**Mean (SD)**	**AL (CR)**
*m*	−2.69 (0.20)	0.82, 69.0%	−2.69 (0.16)	0.63, 56.0%	−2.69 (0.14)	0.58, 47.0%
α	1.80 (0.13)	0.58, 75.5%	1.79 (0.11)	0.44, 58.0%	1.80 (0.10)	0.41, 58.5%
β_1*a*_	0.89 (0.14)	0.55, 85.5%	0.89 (0.10)	0.40, 80.5%	0.90 (0.09)	0.37, 77.5%
β_1*d*_	−1.78 (0.22)	0.90, 85.5%	−1.79 (0.16)	0.66, 78.5%	−1.81 (0.16)	0.62, 74.0%
β_2*a*_	0.90 (0.12)	0.49, 87.5%	0.89 (0.11)	0.36, 78.0%	0.91 (0.09)	0.34, 83.5%
β_2*d*_	0.01 (0.31)	1.24, 95.5%	0.02 (0.23)	0.87, 95.0%	−0.01 (0.22)	0.82, 94.5%
β_3*a*_	0.88 (0.15)	0.58, 85.0%	0.89 (0.11)	0.42, 83.0%	0.88 (0.10)	0.39, 77.0%
β_3*d*_	−0.84 (0.55)	2.20, 95.0%	−0.85 (0.40)	1.56, 93.0%	−0.84 (0.36)	1.46, 94.5%
σ^2^_*A*_	1.11 (0.45)	2.00, 95.5%	1.08 (0.42)	1.56, 93.0%	1.02 (0.32)	1.45, 98.5%
σ^2^_*D*_	0.49 (0.18)	0.80, 94.5%	0.52 (0.20)	0.73, 93.5%	0.57 (0.17)	0.72, 94.5%
σ^2^_*c*_	0.74 (0.19)	0.83, 78.0%	0.72 (0.14)	0.64, 62.0%	0.75 (0.14)	0.62, 70.0%

Under our simulation setting, it appears that the SAS procedures “proc nlmixed” and “proc glimmix” perform unexpectedly poorly especially for binary outcomes. But we have to admit that, although we have explored many options that are available in running “proc nlmixed” and “proc glimmix,” our exploration is surely not exclusive based on our limited knowledge. Besides, we use SAS version 9.3 on our Linux computer system. Recently, a newer version 9.4 of SAS has become available for PC users, which may provide improved performance in fitting the GLMM. Unfortunately, we do not have access to this new version of SAS yet based on our current Linux computer system. The performance of “proc nlmixed” and “proc glimmix” in SAS 9.4 on fitting the re-formulated GLMM needs further exploration.

While the frequencist approach implemented in “proc nlmixed” and “proc glimmix” often require numerical approximations to the full likelihood, the Bayesian approach directly maximizes the full likelihood via MCMC. From our simulation study, it seems that the Bayesian approach can better handle the re-formulated GLMM especially for binary traits. However, it should be pointed out that the choice of priors in using the Bayesian approach could have a significant impact on convergence of an MCMC procedure. Besides the gamma priors for all the τ's, we also tested using uniform priors on σ's and obtained similar results. Throughout our simulation, the Monte-Carlo errors for all the parameter estimates appear to be acceptable. But some auto-correlations in certain Markov chains (e.g., the ones for σ^2^_*D*_ and σ^2^) are noticed. Typically, the auto-correlation could be reduced by thinning the Markov chains. Otherwise, appropriate adjustment is needed in computing the SD of parameter estimates.

## 4. Analysis of T1DGC data for type I diabetes

As an example, we consider fitting a real family data set obtained from the Type 1 Diabetes Genetics Consortium (T1DGC). The data set includes five cohorts: Asia-Pacific (AP), Danish Steno Diabetes Center (DAN), European (EUR), Sardinian (SAR) and United Kingdom Genetic Resource Investigating Diabete (UK). For simplification, we only adopt nuclear families and exclude some grand-parents or grand children (less than 1% of the total subjects). Most of the families (about 80.4%) consist of 2 parents and 2–3 children. There are 13 families that have more than 9 family members, and they all belong to the DAN cohort. The actual numbers of subjects and families we used in the five cohorts are listed in Table 5.

**Table 5:** **Number of subjects and families in T1DGC by cohorts**.

**Cohort**	**AP**	**DAN**	**EUR**	**SAR**	**UK**
No. of subjects	741	664	1936	347	465
No. of families	184	147	475	77	113
Maximum family size	6	14	8	6	6

Our research interest is to test for association of HLA-DQB1 locus with Type 1 Diabetes (T1D) incidence, while appropriately controlling for other potential genetic risk factors on the incidence of T1D. The adjustment for random additive and dominance effects is important because it has been known that T1D is a polygenic disease. Some studies have suggested that other genes such as INS and CTLA4 could be implicated with T1D (Anjos and Polychronakos, 2004; McGinnis et al., 2009). One article “Genetics and Diabetes” from the World Health Organization (WHO) web site “*http://www.who.int/genomics/about/Diabetis-fin.pdf*” also provides a nice review of the T1D. From our previous study (Glisic et al., 2009), we classify the subjects into 4 groups: low risk (DQrisk = 0), moderate risk (DQrisk = 1), high risk (DQrisk = 2), and very high risk (DQrisk = 3) based on the HLA-DQB1 genotypes and CD4 + CD25 + ^*high*^T-cell apoptosis. Gender and age are also known risk factors for T1D. We categorize age into 6 categories: age≤18, 18<age≤30, 30<age≤40, 40<age≤50, 50<age≤60, and age>60. By choosing the “high risk” (the largest group across all cohorts) at HLA-DQB1, male and “age≤18” as a baseline, we fit each cohort separately using the following mixed logistic regression model.

$$\begin{array}{l} \text{logit}P(y_{ij}\text{​}=1|v_{ij},e_{i})=\mu +\alpha_{1}*1_{\left(18<age\leq 30\right)}+\\ \alpha_{2}*1_{\left(30<age\leq 40\right)}+\alpha_{3}*1_{\left(40<age\leq 50\right)}+\\ \alpha_{4}*1_{\left(50<age\leq 60\right)}+\alpha_{5}*1_{\left(age>60\right)}+\alpha_{6}*1_{\left(sex\;=\;F\right)}+\\ \beta_{1}*1_{\left(DQrisk=0\right)}+\beta_{2}*1_{\left(DQrisk=1\right)}+\\ \beta_{3}*1_{\left(DQrisk=3\right)}\text{​}+\text{​}v_{ij}+e_{i} \end{array}$$

where *e_i_* ~ *N*(0, σ^2^_*c*_), *e*^α_*i*_^ (*i* = 1, ···, 5) are odds ratios of having T1D in different age groups comparing with the youngest age group of age ≤18, *e*^β_*j*_^ (*j* = 1, 2, 3) are odds ratios of having T1D in different HLA-DQB1 risk groups comparing with the high risk group, *e*^β_6_^ is the odds ratio of T1D in females versus males, and {*v_ij_*} have the covariance structures as specified in GLMM Equation (1).

To simplify the calculation, we use the expected values to construct the kinship and double coancestry matrices for each family. As the actual kinship and double coancestry matrices should be close to their expected matrices, we would expect only minor deviations from the fitted GLMM. In running “proc glimmix” and “proc nlmixed” to fit the GLMMs, we encountered some severe unconvergence problems (data not shown). In running BRugs + OpenBUGS, we use 10,000 burn-in and another 10,000 updates to estimate the parameters. For the DAN cohort, the current BRugs + OpenBUGS cannot accommodate more than 12 additive or dominant random coefficients in the model specification—see part (2) of Appendix C in Supplementary Material, where “maxsize” (i.e., the number of columns of the stacked matrices *L*_Φ_ and *L*_Δ_) cannot exceed 12 even though the total number of family members can still exceed 12. We also find that some of the parameter estimates become unstable when we actually use 10–12 columns. So we adopt using 8 columns of the stacked matrices *L*_Φ_ and *L*_Δ_ in fitting the Dan cohort. The estimates of odds ratios and variance components in the fitted models from running BRugs + OpenBUGS are summarized in Table 6.

**Table 6:** **Posterior means and 2.5%, 97.5% percentiles of the odds ratios and variance components for type I diabetes in five cohorts of the T1DGC data set**.

**Cohorts**	**AP**	**DAN**	**EUR**	**SAR**	**UK**
Baseline intercept (μ)	2.32 (1.58, 3.29)	2.64 (1.72, 3.67)	3.70 (3.05, 4.48)	1.11 (0.12, 2.14)	2.22 (1.44, 3.20)
18<age≤30 vs. age≤18 (*e*^α_1_^)	1.23 (0.56, 2.82)	0.50 (0.19, 1.39)	0.35 (0.20, 0.59)	0.69 (0.21, 2.32)	0.45 (0.15, 1.35)
30<age≤40 vs. age≤18 (*e*^α_2_^)	0.10 (0.036, 0.25)	0.25 (0.09, 0.64)	0.047 (0.024, 0.086)	0.48 (0.15, 1.45)	0.014 (0.003, 0.045)
40<age≤50 vs. age≤18 (*e*^α_3_^)	0.01 (0.003, 0.04)	0.07 (0.02, 0.19)	0.007 (0.002, 0.014)	0.06 (0.01, 0.22)	0.004 (0.001, 0.013)
50<age≤60 vs. age≤18 (*e*^α_4_^)	0.005 (0.001, 0.022)	0.04 (0.01, 0.11)	0.002 (0.0004, 0.004)	0.009 (0.001, 0.063)	0.005 (0.001, 0.025)
age>60 vs. age≤18 (*e*^α_5_^)	0.012 (0.002, 0.055)	0.009 (0.002, 0.032)	0.0004 (0.0001, 0.0015)	0.003 (0.0001, 0.023)	0.027 (0.0003, 1.25)
Female vs. Male (*e*^α_6_^)	1.27 (0.75, 2.21)	0.60 (0.35, 0.97)	0.60 (0.43, 0.83)	0.60 (0.24, 1.38)	1.36 (0.64, 2.92)
DQrisk = 0 vs. 2 (*e*^β_1_^)	0.09 (0.025, 0.26)	0.02 (0.005, 0.06)	0.04 (0.02, 0.08)	0.11 (0.01, 0.71)	0.02 (0.002, 0.12)
DQrisk = 1 vs. 2 (*e*^β_2_^)	0.14 (0.05, 0.30)	0.30 (0.14, 0.59)	0.25 (0.15, 0.39)	0.19 (0.04, 0.63)	0.24 (0.08, 0.71)
DQrisk = 3 vs. 2 (*e*^β_3_^)	2.26 (1.17, 4.80)	5.84 (2.75, 14.47)	5.91 (3.48, 10.61)	15.89 (4.80, 82.60)	8.36 (3.30, 26.13)
Additive variance (σ^2^_*A*_)	0.71 (0.20, 1.90)	0.69 (0.21, 1.73)	0.47 (0.17, 1.05)	0.68 (0.19, 2.11)	0.71 (0.17, 2.05)
Dominant variance (σ^2^_*D*_)	1.64 (0.28, 6.35)	1.44 (0.21, 4.93)	0.66 (0.23, 1.85)	2.96 (0.41, 10.51)	1.25 (0.28, 4.32)
Family-shared variance (σ^2^_*c*_)	0.62 (0.20, 1.45)	0.66 (0.22, 1.42)	2.36 (1.33, 3.92)	0.56 (0.17, 1.42)	0.63 (0.19, 1.67)

The results have confirmed that age is significantly associated with T1D incidence. In most cohorts except AP, the odds of T1D occurrence reaches the highest in the youngest group of age < 18 and then decreases quickly as age increases. The females appear to have a less chance of having T1D than males in DAN and EUR cohorts. For HLA-DQB1, it appears that in each cohort there is a significant increase of T1D risk in the HLA-DQB1 very high risk group comparing with the high risk group, and the high risk group is also significantly different from the low risk and moderate risk groups after adjusting for the age and gender effects. The odds of T1D for the high or very high risk groups appear significantly higher than that for the low or moderate risk groups, which are consistent with the relative risk of 3–45 of the HLA-DQB1 susceptibility variant reported in “Genetics and Diabetes” from WHO.

Regarding the variance components, the estimates of the additive and family shared variances appear reasonably well, although the dominance variances in SAR and AP cohorts have relatively large variation due to perhaps the lack of information in their family data. To see whether we should not include σ^2^_*D*_ in the models, we also fit the GLMM models without σ^2^_*D*_ and compare them with our previous models. Based on the deviance information criterion (DIC), which is an estimate of the expected predictive error (lower deviance is better) and it can account for both model fitness and model complexity, the models with both additive and dominance variances included have their DIC values of 658.6, 647.3, 1525, 342.2, and 316.6 which are lower than the DIC values of 669.5, 658.9. 1543. 354.1, and 322.3 in the reduced models without σ^2^_*D*_ in AP, DAN, EUR, SAR, and UK cohorts, respectively. So the models with both additive and dominance variances included are preferable. We also estimate the variations contributed by the random putative genetic effects {*v_ij_*}, which account for 22%, 26%, 9%, 27%, and 12% of the total variation in logit*P*(*y_ij_* = 1|*v_ij_*, *e_i_*) for the AP, DAN, EUR, SAR, and UK cohorts, respectively.

The T1DGC family data was collected from the observational retrospective sampling, which likely had over-sampled families with T1D children. It is well known that fitting a mixed logistic regression model with random effects to a retrospective data set may no longer provide the equivalent maximum likelihood estimates of the model parameters as the ones defined in the same model for a prospective cohort. Therefore, the results above are only applicable to the families we obtained from T1DGC. For general populations, an adjustment for the retrospective sampling strategy is needed in order to avoid the bias in the parameter estimates.

## 5. Discussion

In this study, we propose a Cholesky decomposition based re-formulation of the GLMM to fit family data with varied sizes and diverse genetic relatedness. Assuming that there is no genetic correlation between different families, we first apply separate Cholesky decompositions on the genetic kinship (or double co-ancestry) matrices within each family. Next, we stack these Chelosky decomposition matrices from different families into columns to form two stacked matrices. The columns of the stacked matrices can then be treated as fixed covariates with random regression coefficients of equal variances. It should be pointed out that applying separate Cholesky decompositions on each family does not provide computational or storage benefits comparing with the regular Cholesky decomposition on the whole covariance matrix of the random genetic effects because the non-diagonal blocks in the sparse matrix decomposition are actually not saved. However, with the reduced number of columns in our stacked matrices, this re-formulated GLMM can be specified more conveniently using “proc nlmixed” and “proc glimmix” in SAS, or OpenBUGS via R package BRugs. Theoretically, the re-formulated GLMM is equivalent to the original GLMM. Therefore, it should retain the same validity and power in testing the fixed and random genetic effects as the original GLMM. From our simulation, it appears that this re-formulated GLMM can be fitted reasonably well by “proc nlmixed” and BRugs + OpenBUGS for quantitative traits with moderate family sizes. For binary traits, our simulation and real data example shows that at least BRugs + OpenBUGS can appropriately fit the re-formulated GLMM for families of sizes not exceeding 12.

This Cholesky decomposition based re-formulation of GLMM in fitting family data is somewhat analogous to Haseman and Elston's regression method for sib-pairs (Haseman and Elston, 1972). While Haseman and Elston's algorithm regresses the squared sib-pair's phenotypic difference on the kinship and double coancestry coefficients at a targeted locus with fixed effects, our Cholesky decomposition based GLMM regresses all family members phenotypic values on the square roots of the kinship and double coancestry coefficient matrices with random regression coefficients across families.

Except SOLAR, Multic, and *pedigreemm*, many other special software packages are currently available for analyzing pedigree data. For examples, VITESSE implemented the well-known Elston–Stewart's peeling algorithm for computing the likelihood of pedigrees in linkage analysis (Elston and Stewart, 1971; O'Connell and Weeks, 1995). Mendel can run pedigree analysis for quantitative traits (Lange et al., 2013). SAGE SAGE (2012) can be used for pedigree analysis of binary traits. Based on the score statistics and generalized estimating equations, FBAT and its extension PBAT can also be used in association testing for both quantitative and binary traits (Rabinowitz and Laird, 2000; Lange and Laird, 2002; Lange et al., 2004; Laird and Lange, 2006). Nevertheless, our study provides an alternative choice for analyzing the pedigree data by using standard statistical software, which could be useful for statisticians who are not very familiar with these special genetic software packages. The standard statistical software packages often provide many options on choosing different optimization techniques to maximize the likelihood. As long as the optimization procedure converges appropriately, the standard statistical software packages can provide reliable results in most of the cases.

Sometimes we may want to perform hypothesis tests on the existence of certain variance components. For non-Bayesian methods in fitting the GLMM, it has been known that the likelihood ratio statistics (LRS) usually do not asymptotically follow the standard Chi-square distributions under the null because the zeros under the null hypothesis are located on the boundary of the parameter space for the variance components, where the standard regularity conditions no longer hold. As pointed out in Wang et al. (2011), in the hypothesis testing of a single variance component *H*_0_: σ^2^_*A*_ = 0, the asymptotic distribution of LRS has 0.5χ^2^_0_ + 0.5χ^2^_1_ under the null. In the hypothesis testing of more than one variance components such as *H*_0_: σ^2^_*A*_ = σ^2^_*D*_ = 0, the asymptotic distribution of LRS could be a mixture of several chi-square distributions with their weights of the mixture depending on the number of family types. Therefore, directly using LRS to test for the existence of variance components could be incorrect. On the other hand, the Bayesian method can always provide appropriate estimates of the variance components as well as their variances without relying on the asymptotic results.

It should be pointed out that this Cholesky decomposition based re-formulation in fitting the GLMM has some limitations. For example, in order to synchronize the varied family sizes, the number of random effects in smaller families needs to be expanded to match that in the largest family. The Cholesky decomposition also requires that the kinship and double coancestry matrices be positive. When the kinship matrix is calculated from genome-wide genotypes, the kinship or double coancestry matrices could become singular for some of the families. One possible solution to this problem is to apply a different type of decomposition to the kinship and double coancestry matrices for these families. Note that the decomposition matrices can have a reduced number of columns as long as a good approximation to the kinship and double coancestry matrices is maintained. Finally, it appears that the computational speed in fitting the re-formulated GLMM via SAS or OpenBUGs is slow. The proposed GLMM re-formulation is probably more suitable for a refined analysis on certain targeted loci rather than a genome-wide scan for a large number of genetic markers.

### Conflict of interest statement

The authors declare that the research was conducted in the absence of any commercial or financial relationships that could be construed as a potential conflict of interest.
