# Supplementary material for: A re-formulation of generalized linear mixed models to fit family data in genetic association studies
Source: Front Genet. 2015 Mar 31;6:120. doi: 10.3389/fgene.2015.00120 (PMC4379931; doi:10.3389/fgene.2015.00120)
Supplement: Supplementary file 1 [file DataSheet1.PDF]

Supplementary Material: A re-formulation of generalized linear mixed models to fit family data in genetic association studies

Tao Wang [1,*], Peng He [1,2], Kwang Woo Ahn [1], Xujing Wang [3], Soumitra Ghosh [4] and Purushottam Laud [1]

[1] *Division of Biostatistics, Medical College of Wisconsin, WI 53226, USA,*
[2] *Global Biostatistical Science, Amgen, Thousand Oaks, CA 91320, USA*
[3] *Bioinformatics and Systems Biology Core, NHLBI, Bethesda, MD 20892, USA*
[4] *Department of Genetics, Quantitative Sciences, GlaxoSmithKline, King of Prussia, PA 19406, USA*

Correspondence*:
Tao Wang
Division of Biostatistics, Institute for Health and Society, Medical College of Wisconsin, 8701 Watertown Plank Road, Milwaukee, WI 53226-0509, USA, taowang@mcw.edu

APPENDIX A. THE SAS CODE FOR 'PROC GLIMMIX' (r=s=4)

(1) Interpretation of the variables:

- z - adjusted environmental effect
- g - adjusted genetic effect at a targeted marker locus
- e - family shared random environmental effect
- La1, La2, La3, La4 - columns of the stacked kinship coefficient matrix L_Φ
- Ld1, Ld2, Ld3, Ld4 - columns of the stacked double coancestry coefficient matrix L_Δ

(2) SAS code:

```
proc glimmix data=one;
   class famid;
   model y = z g e La1 La2 La3 La4 Ld1 Ld2 Ld3 Ld4
            /dist=normal link=identity;
   random e/subject=famid;
   random La1 La2 La3 La4/subject=famid type=TOEP(1);
   random Ld1 Ld2 Ld3 Ld4/subject=famid type=TOEP(1);
run;
```

APPENDIX B. THE SAS CODE FOR 'PROC NLMIXED' (r=s=4)

(1) Interpretation of the variables:

- z - adjusted environmental effect
- g - adjusted genetic effect at a targeted marker locus
- e - family shared random environmental effect
- La1, La2, La3, La4 - columns of the stacked kinship coefficient matrix L_Φ
- Ld1, Ld2, Ld3, Ld4 - columns of the stacked double coancestry coefficient matrix L_Δ
- a1, a2, a3, a4 - random coefficients of La1, La2, La3, La4
- d1, d2, d3, d4 - random coefficients of Ld1, Ld2, Ld3, Ld4
- va - additive genetic variance component
- vd - dominant genetic variance component
- vc - variance component for the family shared random environmental effect
- ve - variance component for the GLMM model residuals

(2) SAS code:

```
proc nlmixed data=one;
   mean = intercept + alpha*z + beta*g + e
        + a1*La1 + a2*La2  + a3*La3 + a4*La4
        + d1*Ld1 + d2*Ld2  + d3*Ld3 + d4*Ld4;
   BOUNDS va>0, vd>0, vc>0, ve>0;
   model y ~ normal(mean, ve);
   random e a1 a2 a3 a4 d1 d2 d3 d4 ~
     normal( [0,0,0,0,0, 0, 0, 0, 0],
     [vc,
       0, va,
       0, 0, va,
       0, 0, 0, va,
       0, 0, 0, 0, va,
       0, 0, 0, 0, 0, vd,
       0, 0, 0, 0, 0, 0, vd,
       0, 0, 0, 0, 0, 0, 0, vd,
       0, 0, 0, 0, 0, 0, 0, 0, vd]) subject=famid;
run;
```

APPENDIX C. R CODE FOR BRUGS

We consider a data set with 'numfam' families and the total number of subjects is 'n'. Let 'famsize' be the maximum number of family sizes. As an example, we choose famsize=4 (i.e., $r = s = 4$). In general, 'famsize' could vary from 1 up to 12. Variables 'y','z','g1','g2' and 'g3' represent the continuous phenotypic values, environmental covariates and observed genotypes at three targeted marker loci, respectively. 'gd1','gd2' and 'gd3' represent the coded genotypes for dominance effects at the three targeted marker loci. 'La', 'Ld' are n by 4 stacked kinship and double coancestry coefficient matrices for the random genetic additive and dominance effects, respectively. The R code mainly consists of 3 parts.

(1) Main program: preparing the input data set for BRugs:

```
# Interpretation of the variables::
# n - total number of subjects
# numfam - the number of families
# famid - family id
# maxsize - the maximum of family sizes (<=12)
# y - phenotype
# z - environmental covariates
# g - coded genotypes at marker loci
# La - the (n by maxsize) design matrix for the additive
#      genetic variance component
# Ld - the (n by maxsize) design matrix for the dominant
#      genetic variance component

library(BRugs)

# Read in the data and calculate all the variables: "n","numfam",
# "famid","maxsize","y","z","g1","g2","g3","gd1","gd2","gd3","La","Ld".
# Then save them in a file "data.txt":

bugsData(list("n","numfam","famid","maxsize","y","z","g1","g2","g3",
  "gd1","gd2","gd3","La","Ld"),fileName = file.path(getwd(),"data.txt"))
```

(2) A separate file "model.txt" for model specification (e.g., maxsize=4):

```
# Interpretation of the variables:
# a - the random coefficients of the columns in La
# d - the random coefficients of the columns in Ld
# e - family shared random environmental effect
# sigma2.a - additive genetic variance component
# sigma2.d - dominant genetic variance component
# sigma2.c - variance component for family shared environmental effect
# sigma2.e - variance component for model residuals

model {
  for(i in 1:n) {
      Y[i] ~ dnorm(mu[i],tau.e)
      mu[i] <- m+alpha*z[i] + beta1[1]*g1[i] + beta1[2]*gd1[i]
               + beta2[1]*g2[i]+ beta2[2]*gd2[i]
               + beta3[1]*g3[i]+ beta3[2]*gd3[i]
               + v[i] + e[famid[i]]
      v[i] <- a[famid[i],1]*La[i,1]+d[famid[i],1]*Ld[i,1]
             +a[famid[i],2]*La[i,2]+d[famid[i],2]*Ld[i,2]
             +a[famid[i],3]*La[i,3]+d[famid[i],3]*Ld[i,3]
             +a[famid[i],4]*La[i,4]+d[famid[i],4]*Ld[i,4]
      cc[i]<- logit(mu[i])
  }
  vv<- pow(sd(v[]),2)
  mm<- pow(sd(cc[]),2)
  h2<-vv/mm
  for(j in 1:numfam) {
    e[j] ~ dnorm(0,tau.c)
```

```
    for(k in 1:maxsize) {
      a[j,k] ~ dnorm(0,tau.a)
      d[j,k] ~ dnorm(0,tau.d)
    }
  }
  for(j in 1:2) {
    beta1[j] ~ dnorm(0,0.1)
    beta2[j] ~ dnorm(0,0.1)
    beta3[j] ~ dnorm(0,0.1)
  }
  m ~ dnorm(0,0.1)
  alpha ~ dnorm(0,0.1)
  tau.e ~ dgamma(1,1)
  tau.c ~ dgamma(1,1)
  tau.a ~ dgamma(1,1)
  tau.d <- 1/pow(sigma.d,2)
  sigma.d ~ dunif(0,5)
  sigma2.a<-1/tau.a
  sigma2.d<-pow(sigma.d,2)
  sigma2.c<-1/tau.c
  sigma2.e<-1/tau.e
}
```

(3) Main program: run MCMC via BRugs:

```
modelCheck("model.txt")  # recall the model
modelData("data.txt")    # load the data
modelCompile(numChains=1)
modelGenInits()
modelUpdate(10000)
samplesSet(c("m","alpha","beta0","beta1","beta2","sigma2.a",
            "sigma2.d","sigma2.c","sigma2.y","h2","deviance"))
dicSet()   # set deviance
modelUpdate(10000)
dicStats() # display deviance without percentiles
samplesStats("*")
```
